# The Effects of Bupropion on Negative Symptoms in Schizophrenia 

**Published:** 2014

**Authors:** Mojtaba Yassini, Neda Shariat, Mohammad Nadi, Fariba Amini, Mohammad Vafaee

**Affiliations:** a *Shahid Sadughi University of Medical Siences, Yazd, Iran.*; b *Rafsanjan University of Medical Sciences, Rafsanjan, Iran. *

**Keywords:** Schizophrenia, Negative symptoms, Bupropion, SANS

## Abstract

This study was designed determine the efficacy of bupropion versus placebo in subjects with negative symptoms of schizophrenia.

A convenience sample of 40 patients of both genders aged 18-60 years who were living in psychiatric care centers were randomly treated with bupropion (started with 75 mg twice a day; increased to 100 mg thrice daily) or placebo. The diagnosis of schizophrenia was confirmed by a psychiatrist based on Diagnostic and Statistical Manual of Mental Disorders, Fourth Edition, Text Revision (DSM-IV-TR) criteria. Before and after the intervention, severity of negative symptoms was determined using a reliable and valid Persian version of Scales for the Assessment of Negative Symptoms (SANS).

Comparison of post-treatment total SANS score and subscale scores between bupropion treated patients and placebo group demonstrated no significant difference. Moreover, comparison of pre- treatment and post-treatment total SANS score and subscales within 2 groups revealed that nor bupropion neither placebo improved the severity of negative symptoms significantly.

Present study demonstrated that bupropion has no significant effect on SANS score of patients with severe negative symptoms. However, further studies with larger sample size are recommended to achieve more accurate results.

## Introduction

The relative absence of a group of psychological experiences and social interactions in patients with schizophrenia is termed negative symptoms (1). 

These symptoms are very common, and include anergia, apathy, affective flattening, alogia and social withdrawal (2-5). Previous studies have demonstrated significant relationships between negative symptoms and poor premorbid function, lowered IQ, and poor clinical outcome (3, 6-11). 

Since the negative symptoms of schizophrenia impair social and occupational activities (12, 13), the persistence of these symptoms disproportionately limits a patient›s recovery and functions (14, 15). For this reason, increased attention has been given to the treatment of negative symptoms recently (16). 

Although antipsychotic medications are very effective in the treatment of positive symptoms of schizophrenia, negative symptoms are generally poorly responsive to these drugs (17). Therefore, the successful treatment of negative symptoms still remains an important challenge for clinical psychiatrists (18). 

There is increasing evidence on an integrative approach to the pathophysiology of schizophrenia that combines different neurotransmitter systems (19). Based on this evidence, several studies have investigated the role of co-administration of antipsychotics and other psychotherapeutic drugs in the treatment of schizophrenia especially in patients with negative symptoms (17, 20). Various classes of psychotherapeutic medications including antidepressants, buspirone, psychostimulants, anticholinergics or alpha 2 blocking agents have been used to augment the effects of antipsychotic medications in patients with negative symptoms (21-25). However, the results have been contradictory in terms of clinical efficacy (17). 

Bupropion is a strong norepinephrine and dopamine reuptake inhibitor, as well as a nicotinic receptor antagonist. It has been widely prescribed as an atypical antidepressant and a smoking cessation aid. In addition, some studies have investigated the impact of bupropion prescription on negative symptoms of schizophrenia; however, they have reported inconsistent results about its effectiveness (26-28, 37).

In light of the above, this comparative study was designed determine the efficacy of bupropion versus placebo in subjects with negative symptoms of schizophrenia.

## Experimental

*Study population and design:*

After approval of the study by the ethic committee of Shahid Sadoughi University of Medical Sciences (project number :XXXX ) and obtaining informed consent, this double-blind, placebo controlled, randomized clinical trial was performed on patients with chronic schizophrenia who were living in psychiatric care centers. This investigation was performed in Yazd, Iran, between March 2011 and December 2012.

Inclusion criteria were: Patients aged 18-60 who had a history of schizophrenia for at least 2 years, and were treated with a constant dose of typical or atypical antipsychotic agent over 1 month prior to the study were considered eligible. The diagnosis of schizophrenia was confirmed by a psychiatrist based on Diagnostic and Statistical Manual of Mental Disorders, Fourth Edition, Text Revision (DSM-IV-TR) criteria.

Patients with other psychiatric comorbidities -such as mania, major depressive disorder or suicidal tendencies-,patients with organic brain disorders or mental retardation, patient who had any contraindication for bupropion, pregnant women and breastfeeding mothers, alcoholic or drug abuse patients and patients with epilepsy or other significant medical diseases were excluded.

**Figure 1:**
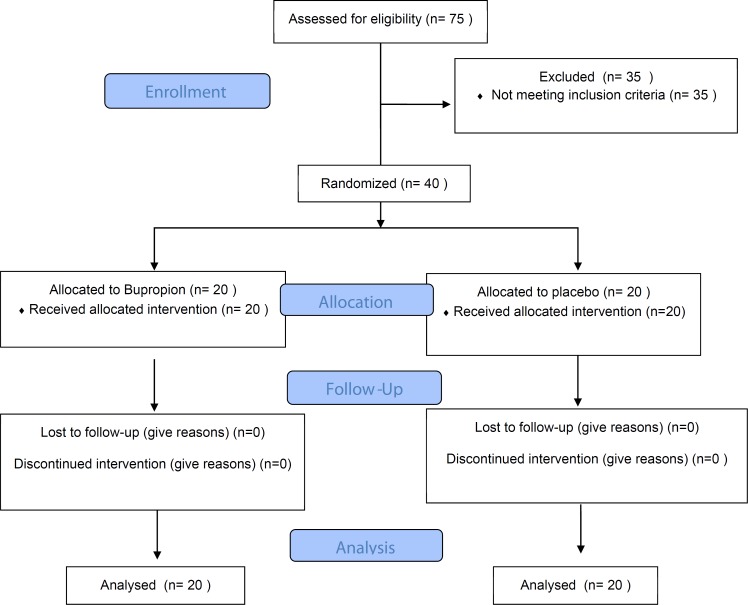
Study flow patients, psychiatrists and other study staff were blinded to the treatment assignment

After initial evaluation of 75 patients, a convenience sample of 40 patients of both genders who met the study criteria were entered the study. Using simple randomization, patients were randomly allocated into 2 treatment groups (Figure 1). 

*Therapeutic regimens*

Patients in the bupropion group (20 participants) were commenced on an initial dose of 75 mg twice a day for the first 3 days, followed by 100 mg thrice a day. In the placebo group (20 participants), an identical placebo tablet was added to their daily medications. Both bupropion and placebo were administered orally.

*Data collection and follow-up*

A reliable and valid Persian version of Scales for the Assessment of Negative Symptoms (SANS) was used to assess the severity of patients’ negative symptoms before the intervention. Using this scale, the severity of negative symptoms including affective flattening or blunting, alogia, avolition-apathy, anhedonia-asociality and attention was evaluated by a psychiatrist. 

All assessments were conducted on a six-point scale (from 0: no symptom to 5: very severe symptom), and a total SANS score and 5 subscale scores were determined. During the first month of the investigation, patients were visited every week; then, every month for 2 months. After 12 weeks of treatment, the SANS score was re-calculated. All 40 participants completed the study. None of patients developed major adverse effects such as acute psychosis after bupropion therapy or significant change in sleep pattern. 

*Statistical analysis*

Data were analyzed by the Statistical Package for the Social Sciences (SPSS) 20.0 (SPSS Inc., Chicago, IL, USA). Independent 

t-test, paired t-test and Chi-square were used when appropriate. p-values less than 0.05 were considered statistically significant.

## Results

There was no significant difference between 2 groups regarding baseline data (Table 1).

Comparison of post-treatment total SANS score and subscale scores between bupropion treated patients and placebo group demonstrated no significant difference. Moreover, comparison of pre- treatment and post-treatment total SANS score and subscales within 2 groups revealed that nor bupropion neither placebo improved the severity of negative symptoms significantly (Table 2). 

**Table 1:** Baseline characteristics of two groups

		**Bupropion ** **(N:20)**	**Placebo ** **(N:20)**	**Total** **(N:40)**	**p-value**
Age (year)		49.58 ± 12.05	49.00 ± 9.45	49.23 ± 10.37	0.88
Sex (male/female)		13/7	12/8	25/15	0.69
Duration of schizophrenia (year)		22.25 ± 12.13	23.71 ± 8.97	22.97 ± 10.07	0.71
SANS score	Total	100.08 ± 11.33	96.11 ± 14.28	97.30 ± 13.56	0.11
	Affect	27.08 ± 3.80	27.50 ± 4.07	27.33 ± 3.90	0.78
	Alogia	20.83 ± 4.68	18.72 ± 3.51	19.56 ± 4.08	0.17
	Avolition	17.16 ± 1.52	15.77 ± 3.55	16.23 ± 3.11	0.23
	Anhedonia	23.58 ± 1.78	21.44 ± 3.43	22.30 ± 3.04	0.12
	Attention	13.33 ± 2.10	11.55 ± 3.61	12.26 ± 3.18	0.13

Data are presented as mean±SD, or number

N: number of patients

SANS: Scales for the Assessment of Negative Symptoms.

**Table 2:** Comparison of pre and post-treatment SANS score (total and subscales) between and within 2 groups

			**Pre-treatment**	**Post-treatment**	**p-value**
SANS score	Total	Placebo (N:20)	96.11 ± 14.28	95.66 ± 13.70	0.76
		Bupropion (N:20)	100.08 ± 11.33	100.67 ± 12.23	0.83
	p-value		0.11	0.31	
	Affect	Placebo (N:20)	27.50 ± 4.07	26.61 ± 3.41	0.35
		Bupropion (N:20)	27.08 ± 3.80	26.83 ± 4.01	0.27
	p-value		0.78	0.87	
	Alogia	Placebo (N:20)	18.72 ± 3.51	18.44 ± 3.16	0.33
		Bupropion (N:20)	20.83 ± 4.68	19.58 ± 4.50	0.10
	p-value		0.17	0.42	
	Avolition	Placebo (N:20)	15.77 ± 3.55	16.01 ± 3.49	0.40
		Bupropion (N:20)	17.16 ± 1.52	17.19 ± 1.23	0.91
	p-value		0.23	0.48	
	Anhedonia	Placebo (N:20)	21.44 ± 3.43	21.12 ± 4.73	0.87
		Bupropion (N:20)	23.58 ± 1.78	23.50 ± 1.45	0.84
	p-value		0.12	0.17	
	Attention	Placebo (N:20)	11.55 ± 3.61	11.44 ± 3.77	0.60
		Bupropion (N:20)	13.33 ± 2.10	13.50 ± 2.39	0.74
	p-value		0.13	0.10	

Data are presented as mean±SD

N: number of patients

SANS: Scales for the Assessment of Negative Symptoms.

## Discussion

Schizophrenia is a chronic debilitating mental illness that consists of severe disturbances in thoughts, cognitions, mood, perceptions, and relationships with others (29). Based on the nature of symptoms, the hallmark symptoms of schizophrenia are mainly categorized into positive symptoms -such as delusions, paranoia, hallucinations and disorganized thoughts- and negative symptoms -including poor motivation, lack of emotional expression, and inability to form appropriate social relationships (28). Negative symptoms are resistant to antipsychotic therapy (30), and have potential impacts on functional outcomes (16). For this reason, various medications have been evaluated to augment the effects of antipsychotic drugs on negative symptoms. It is a common clinical strategy to add antidepressants to the routine antipsychotic regimen of patients with persisting negative symptoms. However, there is still controversy regarding the effectiveness of this method.

Considering the nicotinic receptor antagonist properties of bupropion, it has been widely prescribed for patients with schizophrenia as an aid to smoking cessation (28, 31-34). Meanwhile, there has been a question that whether bupropion can improve negative symptoms by its antidepressant effect or not. However, answers to this question are inconsistent.

This clinical trial demonstrated that adding bupropion to the routine antipsychotic regimen of patients with schizophrenia does not cause any significant improvement of negative symptoms. The effects of bupropion on either total SANS score or subscale scores were not significantly different from those of placebo.

A previous study by Evins *et al. *evaluated the effects of a bupropion (300 mg daily) on smoking cessation of patients with schizophrenia. Meanwhile, they compared bupropion with placebo regarding their effects on negative symptoms (35). They found that bupropion-treated patients had a trend for mean SANS score to decrease from baseline to week 12; however, it was not statistically significant.

George *et al*. also conducted a placebo-controlled trial, and investigated effects of bupropion on smoking cessation and negative symptoms of schizophrenia. Similar to what we have found, they found that bupropion was not significantly more effective than placebo in the treatment of negative symptoms (36). 

On the contrary, Rezaee and colleagues performed a placebo-controlled trial of bupropion for improving the positive and negative symptoms of schizophrenia, and demonstrated that bupropion can improve negative symptoms significantly (28). 

Another pilot study reported significant improvement of SANS score after smoking cessation treatment with bupropion (32). 

Comparing with the present study, subjects of these 2 studies had significantly lower mean of baseline SANS score (37.9 (32) and 11.67 (28) versus 97.30). This difference may be due the different study designs. While the main goal of this study was to evaluate the effectiveness of bupropion in the treatment of negative symptoms, previous studies were aimed to determine the efficacy bupropion in smoking cessation. Therefore, they did not primarily focus on the severity of negative symptoms. In addition, in order to have close supervision on patients regarding taking medications regularly, this study was performed on patients who were living in the psychiatric care centers who have more severe mental disorders.

Although Eden Evis *et al*. excluded patients with depression (32), Rezaee *et al*. did not consider depression as an exclusion criterion (28). Therefore, the improvement that they have observed could be indirectly caused by antidepressant effects of bupropion on concurrent depression. When patients with depressive disorders are not excluded, and both positive and negative symptoms are investigated, it is unclear whether the effect of bupropion on the negative symptoms is not merely secondary to the reduction of depression or of positive symptoms (23).

*Limitations*

This study was performed on a relatively small sample size. Comparing with previous studies, our patients were suffering from more severe mental disorder that may be a probable cause for failure of treatment. 

In summary, we conclude that bupropion has no significant effect on SANS score of patients with severe negative symptoms. However, further studies with larger sample size are recommended to achieve more accurate results. Moreover, given these differences and in order to avoid probable effects of negative symptom severity, it would be helpful to re-evaluate effects of bupropion after grouping patients according to the severity of negative symptoms. 
